# Hearing (rivaling) lips and seeing voices: how audiovisual interactions modulate perceptual stabilization in binocular rivalry

**DOI:** 10.3389/fnhum.2014.00677

**Published:** 2014-09-04

**Authors:** Manuel Vidal, Victor Barrès

**Affiliations:** ^1^Laboratoire de Physiologie de la Perception et de l'Action, UMR7152 Collège de France, CNRSParis, France; ^2^Institut de Neurosciences de la Timone, UMR7289 Aix-Marseille Université, CNRSMarseille, France

**Keywords:** binocular rivalry, volition, selective attention, audiovisual integration, speech perception, McGurk effect

## Abstract

In binocular rivalry (BR), sensory input remains the same yet subjective experience fluctuates irremediably between two mutually exclusive representations. We investigated the perceptual stabilization effect of an additional sound on the BR dynamics using speech stimuli known to involve robust audiovisual (AV) interactions at several cortical levels. Subjects sensitive to the McGurk effect were presented looping videos of rivaling faces uttering /aba/ and /aga/, respectively, while synchronously hearing the voice /aba/. They reported continuously the dominant percept, either observing passively or trying actively to promote one of the faces. The few studies that investigated the influence of information from an external modality on perceptual competition reported results that seem at first sight inconsistent. Since these differences could stem from how well the modalities matched, we addressed this by comparing two levels of AV congruence: real (/aba/ viseme) vs. illusory (/aga/ viseme producing the /ada/ McGurk fusion). First, adding the voice /aba/ stabilized both real and illusory congruent lips percept. Second, real congruence of the added voice improved volitional control whereas illusory congruence did not, suggesting a graded contribution to the top-down sensitivity control of selective attention. In conclusion, a congruent sound enhanced considerably attentional control over the perceptual outcome selection; however, differences between passive stabilization and active control according to AV congruency suggest these are governed by two distinct mechanisms. Based on existing theoretical models of BR, selective attention and AV interaction in speech perception, we provide a general interpretation of our findings.

## Introduction

In everyday life, phenomenal experience builds upon evidence about the outside world provided by our senses combined with internal expectations. In most situations, this produces various more or less plausible interpretations of what is out there, yet only one will eventually emerge to create conscious perception. A typical way to investigate this mechanism is to use bistable stimuli for which only two mutually exclusive interpretations compete. Binocular rivalry (BR) refers to the situation in which each eye receives one of two incompatible images. After a variable transition period in which fusion is attempted, the observer experiences inevitable perceptual alternations between the two images. Volitional control in BR is defined as the capacity to mentally interfere with the outcome of the competition between the two possible percepts. While selective attention studies have been classically devoted to competing items located in different spatial positions, BR addresses the situation where the competing items are spatially superimposed. Despite this conceptual difference, recent findings suggest that the same brain area, the intraparietal sulcus (IPS) known to host spatial maps and saliency filters involved in attentional control, would also be at the core of the perceptual selection process in BR (Zaretskaya et al., 2010). Selective attention can modulate rivalry in different ways: determining the initial percept (Chong and Blake, 2006; Klink et al., 2008), increasing the alternation rate (van Ee et al., 2005), or prolonging dominance durations (Paffen et al., 2006; Alais et al., 2010). Lastly, selective attention can bias perceptual alternations in order to promote one of the rivaling percepts by maintaining it dominant as long as possible (Chong et al., 2005) and switch back to it when suppressed (Kanai et al., 2006). In this project, we used rivaling natural videos of moving lips to assess how a relevant voice sound interferes with the selection of the visual outcome.

If an observer experiences a perceptual competition in one modality, information in another modality can interact with the perceptual alternation dynamics in different ways. If this information is congruent with one of the competing percepts, it could strengthen the saliency of that percept and extend its dominance periods compared to the other, even for passive observation. Several studies investigating how auditory information could bias BR reported evidence of such passive interactions. Sound amplitude modulated in synchrony with the contrast (Kang and Blake, 2005) or associated with the spatial frequency (Guzman-Martinez et al., 2012) of one the rivaling gratings, sound moving consistently with one of the competing visual dot-field motion direction (Conrad et al., 2010), or sound evoking semantically one of the rivaling pictograms (Chen et al., 2011) will all increase the corresponding stimulus dominance. Conversely, vision can also interact passively with an auditory bistability. Adding a synchronized video of lips talking will increase the perceptual stability of the congruent interpretation in the verbal transformation effect (Sato et al., 2007). Other modalities have also proved to bias visual competition in favor of the congruent rivaling percept: grating-shaped tactile information promotes the visual perception of the aligned grating when two orthogonal gratings are rivaling (Lunghi et al., 2010; Lunghi and Alais, 2013), smell promotes the associated visual item when rivaling with another one (Zhou et al., 2010). If the stimuli involves a low-level audiovisual (AV) integration or if one of the rivaling visual stimuli simply cannot integrate with the sound, such limited congruence might not be strong enough to yield significant effects on the competition dynamics. Adding a looming sound to rivaling animations—a looming bullseye pattern vs. a rotating radial pattern—did not increase the dominance of the looming visual percept (van Ee et al., 2009). Adding voice to a dynamic version of the face/vase illusion in which the lips are moving consistently did not really favor the dominance of the face percept (Munhall et al., 2009). Finally, if the additional modality is totally unrelated to both competing percepts, then perceptual destabilizing effects seem to occur. For instance, when viewing the motion quartet illusion, transient sounds or flashes increase the alternation frequency of the current perceptual interpretation (Takahashi and Watanabe, 2011). Interestingly, it is possible to modulate these alternations by presenting sounds that have been previously associated to each interpretation, using an implicit learning procedure, even though sounds were perceptively undistinguishable (Takahashi and Watanabe, 2010).

The additional information provided in another modality can also modulate the volitional control over the perceptual competition. To our knowledge, only two studies have addressed this issue. In the first, mentioned above (van Ee et al., 2009), although the looming sound had no influence on perceptual dominance of the passively viewed rivaling animated patterns, it significantly improved the capacity to promote the looming bullseye percept. Other manipulations evidenced that: explicit attention to the additional looming sound is required; desynchronizing the looming sound using a different frequency cancels the effect; using just tones synchronous with either the looming bullseye or the rotating disk is enough. The use of low-level visual stimuli with such artificially matching sounds could have excluded the possibility to observe passive interactions and limited the required congruence to temporal features. More recently, the influence of a semantically congruent soundtrack (singing birds or car engine) on the perceptual dynamics of BR (bird or car pictogram) was investigated (Chen et al., 2011). Listening to either soundtrack improved slightly but significantly, the capacity to hold the congruent visual percept compared to the incongruent one (about 6% in average of dominance time over the trial). This improvement led to a smaller difference in dominance time than when participants listened passively to the congruent soundtrack (about 10%). Furthermore, the size of the effect is rather small when contrasted with the difference between the volition and passive conditions (about 18%). Taken together, these findings suggest that the strong volitional effect is only slightly modulated by the congruence effect, as observed with passive viewing. This could stem from the congruence between soundtrack and visual pictures that was limited to higher-level semantics, reducing the possibility of low-level sensory integration to enhance voluntary control.

In a series of experiments, we investigated the effect of adding the voice /aba/ on the visual competition between two looping videos of lips uttering /aba/ and /aga/, respectively. This effect was tested in two situations: when experiencing passively the visual rivalry or when exerting a top-down volitional control to promote one of the percepts. We used speech stimuli, known to involve interactions between visual and auditory cortices at various processing stages, in order to increase the range of action of top-down attentional control (Talsma et al., 2010) and see whether we can achieve stronger effects than what was reported in the literature with lower-level stimuli. We selected for this project participants highly sensitive to the McGurk effect (McGurk and MacDonald, 1976) so that when the /aga/ viseme won the competition, they would almost systematically hear the illusory /ada/ and not /aba/. Thereby, we could assess how speech auditory information modulates the perceptual dominance of visemes whose AV congruence is either real or illusory. Participants continuously reported the lips color of the dominant percept for rivaling static images of mouths (Experiment A), rivaling videos of talking lips without sound (Experiment B) or with the voice of one of the videos (Experiment C). In order to assess the volitional control, in a number of trials participants were instructed to hold as long as possible one of the two rivaling percepts. The level of AV matching was manipulated by comparing the physically congruent condition (/aba/ viseme with /aba/ voice) with the subjectively congruent condition resulting from a perceptual illusion (/aga/ viseme with /aba/ voice). To summarize, we predict that the additional voice will have the following effects on the perceptual alternation dynamics. For passive observation, when adding the voice, we expect to find an increased perceptual stability of the physically matching /aba/ viseme (real congruence), and to a lesser extent of the subjectively matching /aga/ viseme (illusory congruence). Similarly, when trying to hold the viseme /aba/ that physically matched the voice we expect to find a strong volitional control, but not so much when trying to hold the /aga/ viseme that only matched subjectively.

## Methods

### Apparatus

#### Raw video material

The main material for this series of experiment was two raw video sequences shot at 25 fps of a woman uttering the sound /aba/ or /aga/. Sequences lasted 41 frames (1640 ms) and were cut so that the consonants /b/ and /g/ started at the exact same frame position (760 ms). The AV asynchrony of the /aba/ voice dubbed over the /aga/ video sequence was therefore below one frame (40 ms), which falls within the temporal window of integration of the McGurk effect (van Wassenhove et al., 2007). The final and first frames were very similar so that a minimal cross-fade of two frames was enough to avoid noticeable jerks when looping the videos.

#### Visual stimulation

**Figure 1:**
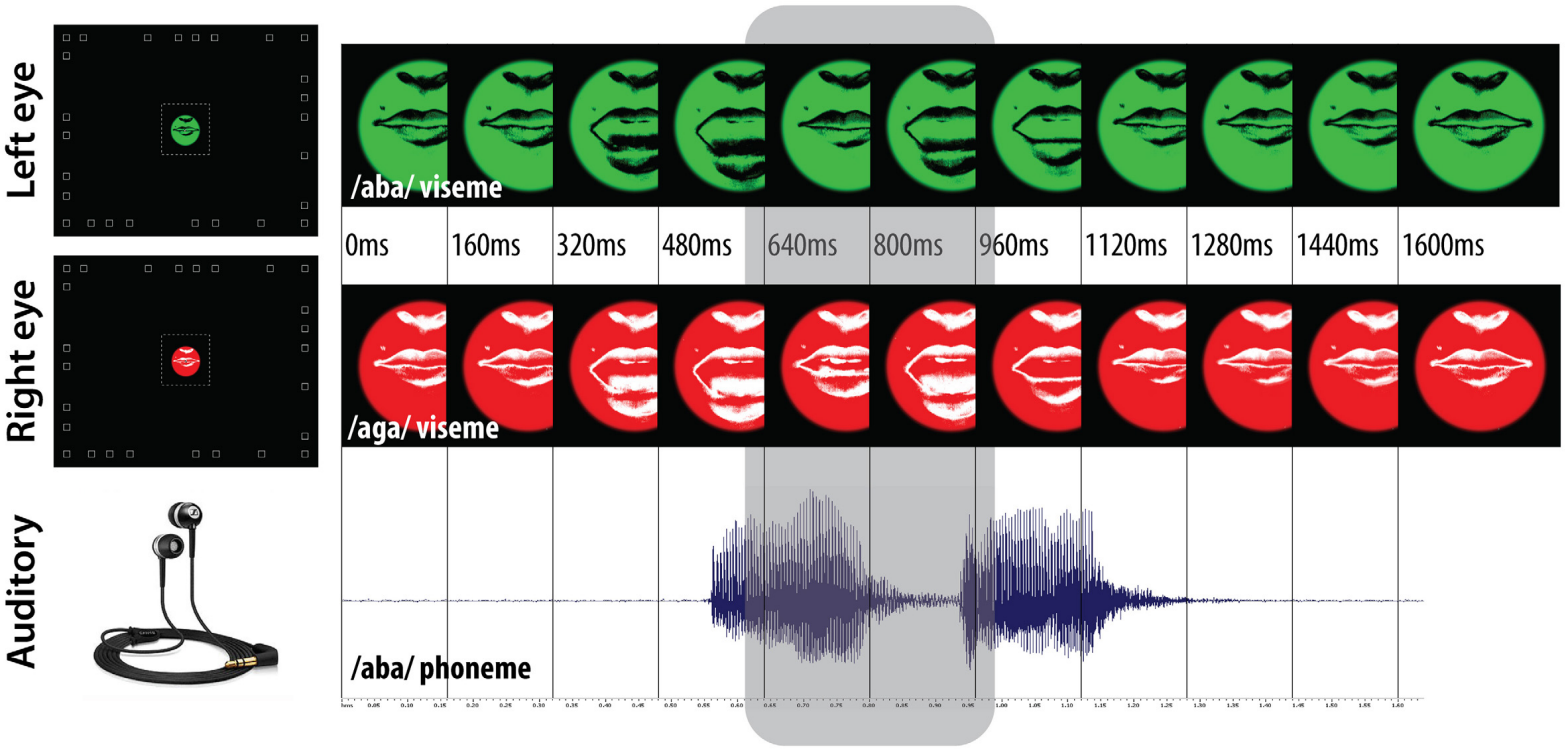
**Audiovisual stimulations**. A pair of video sequences or static snapshots were displayed to the left and right eye using the NVisor SX dual-channel head mounted display, and sound was played using the Sennheiser CX300-II ear canal phones. The color filtering used (black/white lips on a green/red background) enabled rivalry of the spatially superimposed videos displayed within 5° circular apertures. The random disposal of white squares in peripheral vision was used to ensure proper binocular alignment. The time-course of /aba/ and /aga/ viseme video sequences is presented together with the /aba/ phoneme wave-sound, the gray area highlights the time interval during which the two visemes diverge.

The two raw video sequences (lips uttering /aba/ and /aga/) were processed. Lips features were emphasized using contrast filters and two different color filters were applied so that two video sequences were created for each lip motion: one with black lips on a green background and another with white lips on a red background (see the snapshots of the rivaling videos in Figure 1). The color filters were designed in order to produce rivaling stimuli when applying each filter—red or green—to each of the two eyes. It is important to notice that the rivaling properties of the videos stem from the color filtering and not from the content discrepancy. Indeed, the exact same visual images can rival with this technique. Static snapshots were taken at the very beginning of the lips uttering /aba/ videos, one for each color filter, in order to measure individual baselines of the static rivalry dynamics.

The visual stimulations were presented using the dual channel Head Mounted Display (HMD) nVisor SX, running at 60 Hz with a resolution of 1280 × 768 and covering a field of view of 44° × 34°. The stimuli (videos or static images) were displayed in central vision through a soft-edged 5° circular aperture, optically located at an infinite distance (accommodation and vergence). The size of the aperture was determined as a tradeoff between low piecemeal levels in rivalry (smaller size) and good visibility of the lips' motion (bigger size). Since stimulus intensity can bias the competition for dominance, we minimized the intensity difference of our rivaling stimuli by matching their average luminance. The luminances of the red (12 cd/m^2^) and green (21 cd/m^2^) backgrounds were determined in order to have the same average luminance (15 cd/m^2^) when combined with the lips (white lips with green and black lips with red). Binocular fusion was facilitated in every trial by a random disposal of white squares in peripheral vision in the otherwise black background (a fixed random distribution on a 40° × 30° rectangle with a null disparity, square size ~1°). In BR, the propensity of perceptual flips increases just after saccades, though these cannot be used to exert voluntary control on the perceptual outcome (van Dam and van Ee, 2006). In order to limit such biases on the competition dynamics, during the entire trials a fixation cross was displayed at the center of the circular aperture (black and white checker in a 0.3°-diameter disk), falling roughly at the junction between the two lips when the mouth was closed. It also helped binocular alignment and keeping attention focused on the lips.

#### Auditory stimulation

The auditory stimulation was played using Sennheiser ear canal phones. In the experimental conditions with sound, the soundtrack of the video of lips uttering /aba/ was used (see the sound wave in Figure 1). It is important to notice that the video sequences were constructed such that this soundtrack was synchronized with both video sequences, and that the sound and video sequences were triggered synchronously at the beginning of each loop preventing any possible drift between both. The soundtrack of the video of lips uttering/aga/ was never used.

#### Keyboard responses

The left and right arrows of a keyboard were used to record the participant's responses. Each key was associated to a given percept (black or white lips) and was kept constant throughout the experimental series. Participants were trained to keep the key pressed as long as the corresponding percept was stable, and to release it as soon as a noticeable change occurred (alternation to the other percept or piecemeal).

### Procedure

#### Participants

Twelve subjects (8 men, 4 women) all right handed participated in the entire experimental program. They were aged between 20 and 31 years old (average of 25.8). All were naïve to the purpose of the experiment and to the McGurk effect.

#### Ethics

All participants gave a written consent after being informed of the experimental methods used and their right to interrupt the experiment whenever they felt like. This project was approved by the regional ethics committee (*Comité de Protection à la Personne de l'Île de France*, with the authorization number 10013), and complies to the regulations described in the Declaration of Helsinki.

#### Experimental program

**Figure 2:**
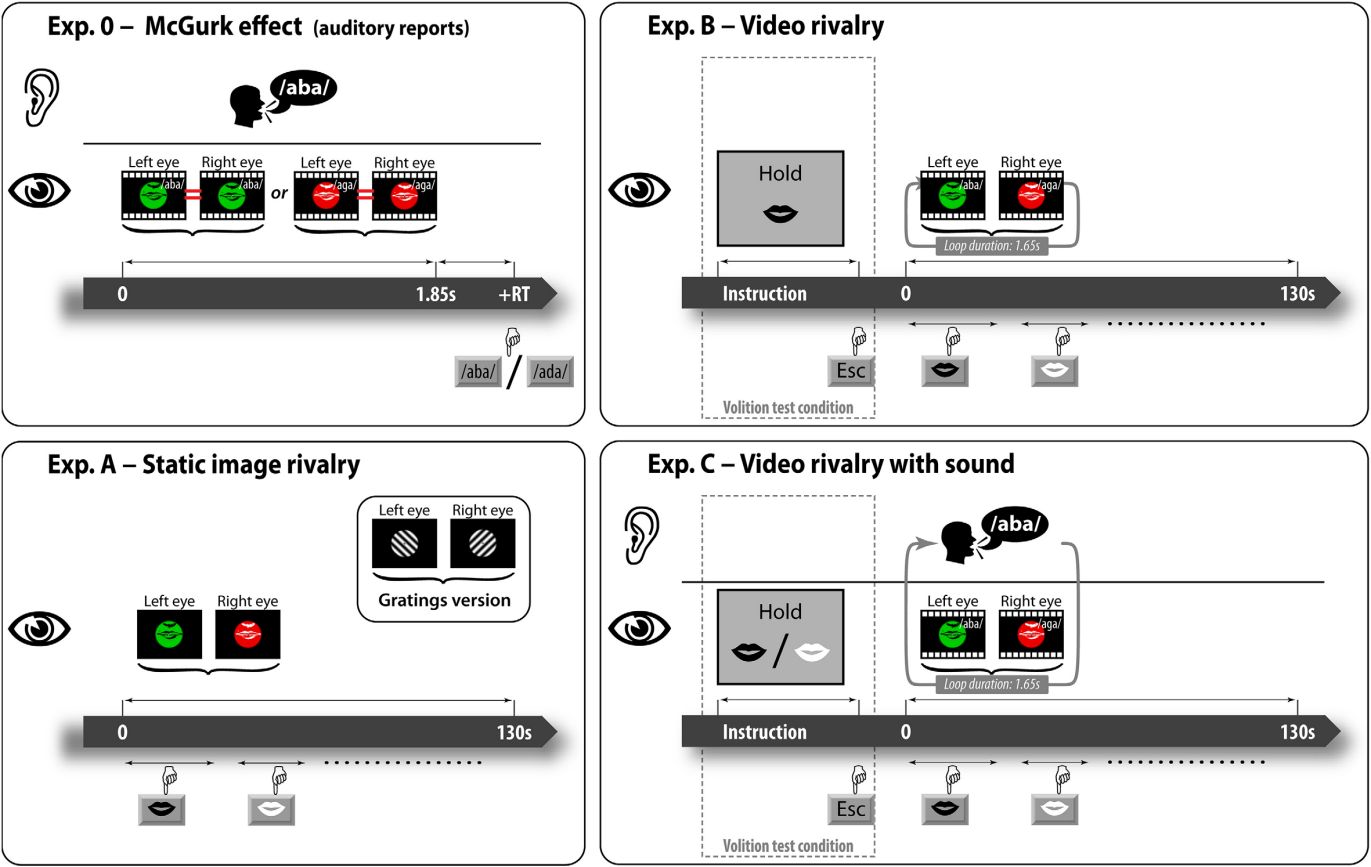
**Trial timelines of the experiments**. In the preliminary experiment, subjects viewed single sequences of videos (no rivalry here) synchronized with the /aba/ voice and then selected the auditory percept (either /aba/ or /ada/). In the main experiments, subjects reported continuously during 130 s trials the currently seen percept (lips color) for: rivaling static images (Experiment A), looping rivaling videos either without sound (Experiment B), or with the /aba/ voice (Experiment C). In some trials of Experiments B and C, subjects were instructed to hold as long as possible the face percept with either black or white lips.

The experimental program consisted in a series of three experiments designed to investigate the perceptual dynamics of BR for rivaling static images (Experiment A), rivaling videos (Experiment B) or rivaling videos with synchronized sound (Experiment C). We conducted a preliminary test (Experiment 0) that measured individual sensitivity to this McGurk effect in order to retain only highly sensitive subjects (further details are found in the Supplementary Material Section 1). Each subject was tested on the entire program in the exact same experimental sequence (0, A, B, C). Figure 2 shows the trial timelines of the different experiments. The general structure of trials was very similar: a pair of rivaling stimuli (black lips vs. white lips) is presented one to each eye for 130 s and the task is to report continuously the dominant percept (lips color) by pressing specific keys.

In the static image rivalry Experiment (A), the visual stimulation was either a pair of static gratings (+45° vs. −45° orientation) or the pair of rivaling lips snapshots (black lips vs. white lips, see Visual stimulation). The gratings were constructed using a sinusoidal luminance wave function with maximal contrast with a spatial frequency of 1 cycle/degree. In the gratings condition, the left-tilted grating was always presented to the left eye and the right-tilted grating to the right eye. In the static lips condition, the black lips were always presented to the left eye and the white lips to the right eye. The experiment was composed of seven continuous report trials, three with the gratings condition followed by three with the static lips condition. The last trial used the simulated rivalry condition in which the same lips image was presented in both eyes (no rivalry) and the images were switched by the computer after a random duration drawn from a Gaussian distribution (*m* = 3 s, σ = 2 s). We designed this last condition to control for correct report of perceptual alternations and to measure individual reaction times. Subjects could have a short break after each trial and a longer one after each block (minimum of 5 min). Subjects started by a four trials training session, each trial lasting only 60 s. The first two used the gratings condition and the last two the static lips condition. The entire experiment lasted about 30 min. The grating stimuli were tested to provide a baseline for each participant in order to check for good rivalry dynamics when using our new stimuli.

In the video rivalry Experiment (B) the video sequences were used without soundtrack. In each trial, a pair of rivaling videos was looped during 130 s while subjects had to report continuously the currently seen lips color. The black lips videos were always presented to the left eye and the white lips videos to the right eye. In each pair of rivaling videos used, the lips were uttering a different phoneme (/aga/ vs. /aba/ visemes) though without soundtrack. In this experiment, the volitional capacity of subjects was assessed. In the passive condition, we instructed subjects to report the lips color perceived without thinking or making any effort to promote any of the two rivaling percepts. No particular message was displayed before the trial start. In the volition test condition, subjects were instructed to try to hold as long as possible the black lips percept, which was associated with the /aba/ viseme. A message indicating to hold black lips was displayed before the trial start. Subjects knew that to do so they could try prolonging the black lips viseme's duration but they could also try shortening the other viseme's duration. Since it is impossible to prevent the perceptual alternations, we instructed them to primarily pay attention to the main task that consists in reporting these alternations. The experiment was composed of 9 continuous report trials grouped in blocks of three trials: the first two with the passive condition and the last one with the volition test condition. The black color being always presented to the left eye, the two associations between viseme (/aba/ or /aga/) and eye were tested in the passive conditions of each block, the order of which was balanced across subjects. Subjects could have a short break after each trial and a longer one after each block (minimum of 5 min). The experiment started with a training session of three trials each lasting 60 s in which the condition order followed that of a typical block. The entire experiment lasted about 45 min.

In the video rivalry with sound Experiment (C) the video sequences were used synchronously with the /aba/ soundtrack. This experiment is analogous to the previous one with a few modifications. The /aba/ soundtrack was always played synchronously with the looping rivaling videos (/aba/ vs. /aga/ viseme). In this experiment, two types of volition conditions were tested: the hold /aba/ condition in which subjects were asked to promote the black lips percept associated with the /aba/ viseme, and the hold /aga/ condition in which subjects were asked to promote the white lips percept associated with the /aga/ viseme. The experiment was composed of 12 continuous report trials grouped in blocks of 4 trials: the first two with the passive condition and the last two with the volition test conditions (hold /aba/ and hold /aga/). The order of the last two was also balanced across subjects. Subjects could have a short break after each trial and a longer one after each block (minimum of 5 min). The experiment started with a training session of three trials each lasting 60 s, two with the passive condition and one with the hold black lips condition. The entire experiment lasted about 45 min. In order to minimize the habituation effects observed after long exposures to BR stimulations, for each participant this Experiment (C) was conducted after a minimum of 3 days following the previous Experiment (B).

### Data analysis

Subjects reported continuously the rivalry oscillation dynamics by pressing the keyboard button corresponding to the current perceptual state. From this report, each trial resulted in a set of dominance durations associated to each of the two rivaling stimuli (time intervals in which the perceptual outcome is clearly defined by the given stimulus) and a set of piecemeal durations (none of the keys down). This data was corrected following rules described in the Supplementary Material Section 2 in order to remove initial and final reports, to suppress very short piecemeal durations reported between two identical perceptual states, and to adjust successive dominance durations when overlapping. Raw dominance durations follow a gamma probability distribution (Supplementary Material Section 3.3) therefore plots as well as statistical analysis will be based on the log value of these dominance durations (normal distribution). Lastly, although this is often overlooked in the literature, dominance durations are not sufficient to describe the perceptual dynamics of BR. For instance, changes in piecemeal/percept dominance repartition over the trial or in flip rate cannot be properly captured through dominance durations analyses alone. In order to better grab the subtle changes in the perceptual dynamics, we also analyzed the dominance fraction of each percept (including piecemeal) for each trial. We computed each percept's fraction by summing all valid dominance durations of that percept within the trial divided by the total effective duration of that trial (which corrects for the border effects, as described in the Supplementary Material Section 2). Statistical analyses included ANOVAs, student *t*-tests and Tukey's HSD test of significance for *post-hoc*s.

## Results

In a preliminary experiment, we measured the individual sensitivity to the McGurk effect. We kept for the experimental program only 12 highly sensitive subjects for whom AV fusion occurred almost systematically (41.4% of participants from our initial set, further details in the Supplementary Material Section 1). All the conditions tested (gratings, static lips, talking lips with and without sound, either passively or holding one of the percepts) verified the three characteristics of BR (perceptual outcome exclusivity, standard stochastic component of dominance durations and inevitable perceptual alternations, Supplementary Material Section 3). Finally, the two color associations resulted in similar intensity regarding the competition for dominance (Supplementary Material Section 3.5). Therefore, the effects reported below are largely independent of the viseme/color association and stem only from the different interactions between the voice and the lips' motion. We also checked that the lips' motion did not affect significantly the competition dynamics (Supplementary Material section 3.4). To our knowledge, this is the first successful design of animated BR using post-processed natural videos.

### Passive visual rivalry when adding sound

**Figure 3:**
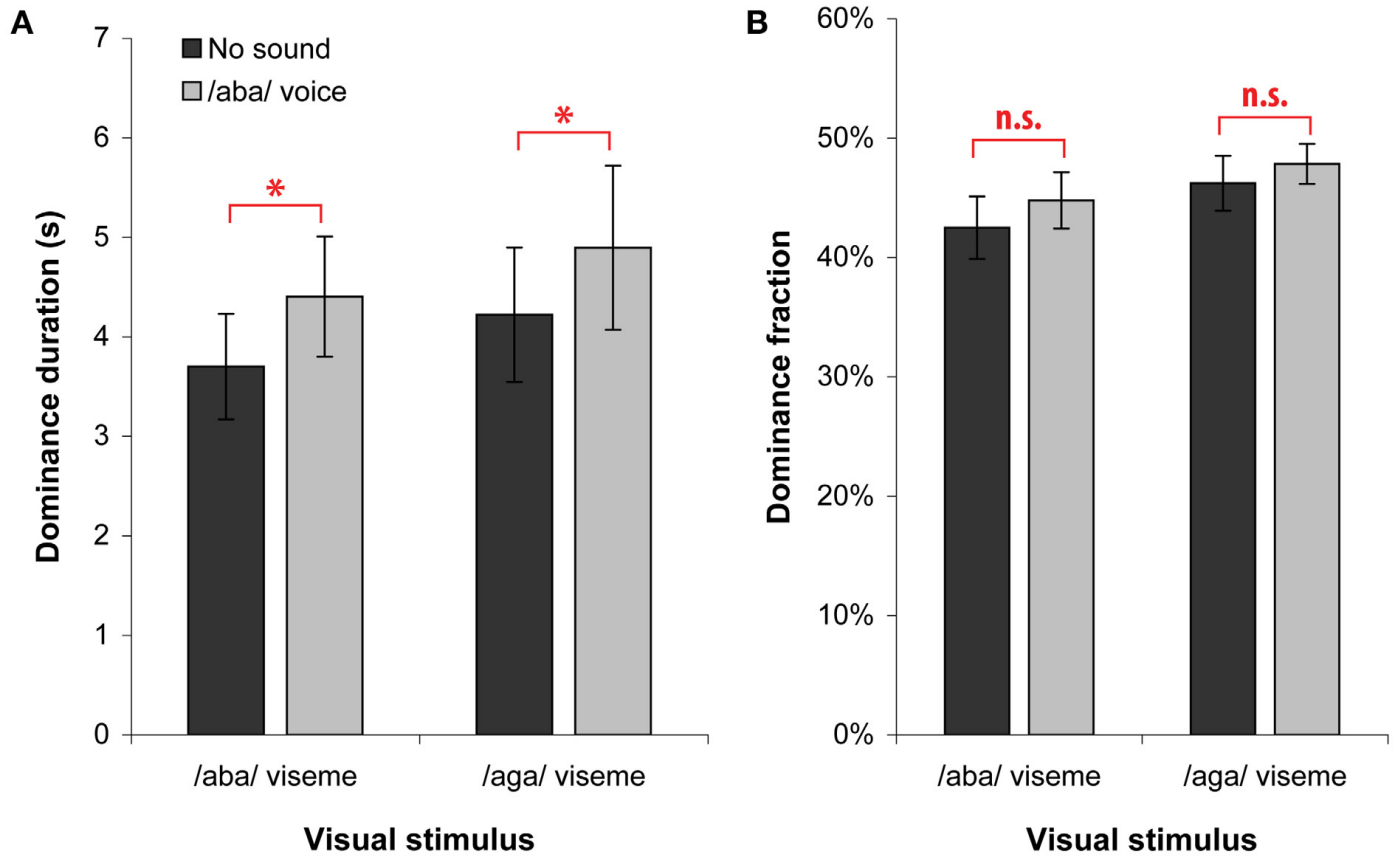
**Dominance duration (A) and fraction (B) when viewing passively**. Dominances are plotted according to the perceived viseme (/aba/ and /aga/) for both the *No sound* condition (from Experiment B) and the */aba/ voice* condition (from Experiment C). We found a significant increase of dominance durations that was independent of the congruence between the currently seen viseme and the voice. Asterisks indicate significant differences and error bars indicate standard errors of the mean.

In passive trials, we asked participants to report continuously the color of the lips in the dominant video without making any effort to control the perceptual outcome. We measured the effect of adding the /aba/ voice sound by comparing the condition with sound of Experiment C (gray bars) and the condition without sound of Experiment B (dark bars, see Figure 3). The overall dominance durations showed an increase of 690 ms [*F*_(1, 11)_ = 4.9; *p* < 0.05] and *post-hoc* tests showed that this increase was significant for each of the two visemes (both *p* < 0.001). There was no significant change in dominance fraction, as expected considering that both rivaling percepts were similarly affected by the addition of sound and that the level of piecemeal remained constant (mixed percept).

Adding the /aba/ voice resulted in an increased dominance durations for both visual percepts. In other words, the voice slowed down the rivalry alternation rate. For our participants who proved to fuse systematically the /aba/ voice with either the /aba/ or /aga/ moving lips, both the /aga/ percept (McGurk fusion) and the /aba/ percept (direct fusion) have increased stability periods during which they win the competition. This increased stability does not require real congruence between the auditory and visual information. This finding extends to AV speech the findings reported earlier for gratings modulated by an additional sound (Kang and Blake, 2005; Guzman-Martinez et al., 2012), moving dots congruent with the direction of a moving sound (Conrad et al., 2010) or images semantically congruent to a background sound (Chen et al., 2011).

### Volitional control of visual rivalry when adding sound

**Figure 4:**
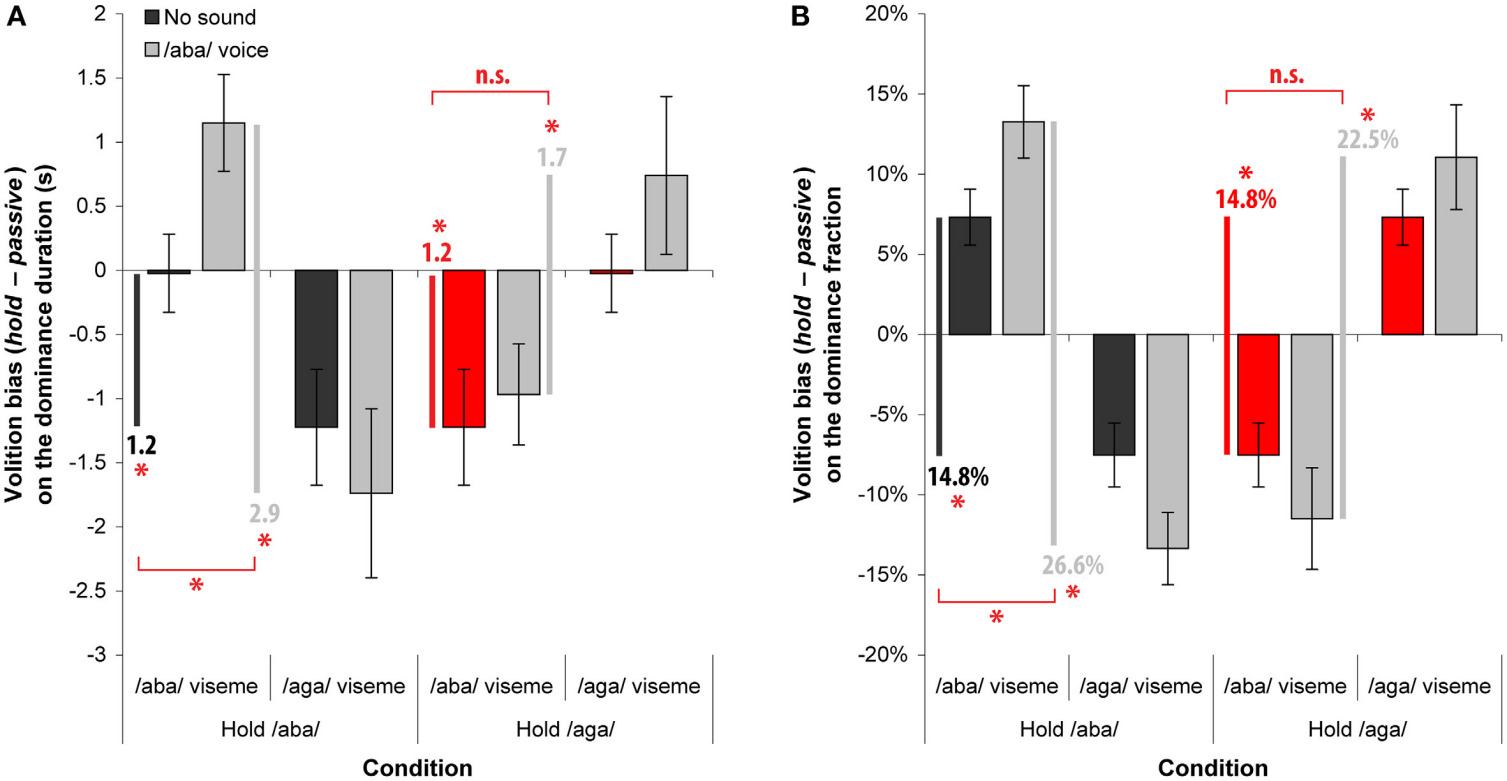
**Volition biases on the dominance duration (A) and fraction (B)**. For each visual percept, the *volition bias* is the difference in performance between the volition condition and the associated passive condition. Each plot shows the volition biases obtained without sound (from Experiment B) and with the /aba/ voice (from Experiment C) for each rivaling viseme when holding /aba/ or when holding /aga/. Red bars indicate the reference used without sound for the hold /aga/ condition corresponding to the symmetrical effect observed in Experiment B (see text for more details). Dark and light gray lines and the values below indicate the *volitional control strength* defined as the volition bias difference between the viseme to hold and its rival (/aba/ – /aga/ for the first two conditions and /aga/ – /aba/ for the last). Asterisks indicate significant differences and error bars indicate standard errors of the mean.

In order to assess participants' volitional control, in some trials they were instructed to promote a particular visual percept by attempting to hold it as long as possible when dominant and to switch back to it when suppressed, while monitoring and reporting the inevitable perceptual alternations. Selective attention could therefore bias the dominance of both /aba/ and /aga/ viseme. We define the *Volition bias* as the difference in performance (dominance duration or fraction), measured separately for each viseme, between the active condition when trying to hold the target viseme and the passive observation. This volition bias should be positive for the target percept to hold and negative for the other. We then define the *volitional control strength* as the combination of the volition biases observed for the target viseme to hold and the other. For instance, in the hold /aba/ condition, it corresponds to the difference between the volition bias on the /aba/ viseme with that on the /aga/ viseme. Figure 4 shows subjects' average volition biases for dominance durations log (left) and fractions (right). These are plotted according to the volition task (hold /aba/ or hold /aga/) and dominant viseme (/aba/ or /aga/) for both the *No sound* condition (from Experiment B) and the */aba/ voice* condition (from Experiment C).

Table 1 presents the volition biases (hold condition – passive condition) and the volition strengths (bias on the viseme to hold – bias on the other viseme) observed for the dominance duration and fraction, together with the corresponding statistical comparisons.

**Table 1:** **Dominance duration and fraction (mean ± SE and statistical effects)**.

	**No sound**			**/aba/ voice**						**Volition strength comparisons**	
	**Hold /aba/ (1)**			**Hold /aba/ (2)**			**Hold /aga/ (3)**				
	/aba/*	/aga/*	**/aba/ – /aga/**	/aba/*	/aga/*	**/aba/ – /aga/**	/aba/*	/aga/*	**/aga/ – /aba/**	**(2) vs. (1)**	**(3) vs. (1)**
Duration	0.0 ± 0.30 s n.s.	**−1.2 ± 0.45 s *p* < 0.004**	**1.2 ± 0.27 s *F*_(1, 11)_ = 14.1; *p* < 0.005**	1.2 ± 0.38 s *p* = 0.052	**−1.7 ± 0.66 s *p* < 0.002**	**2.9 ± 0.75 s *F*_(1, 11)_ = 33.4; *p* < 0.001**	−1.0 ± 0.39 s n.s.	0.7 ± 0.61 s n.s.	**1.7 ± 0.88 s *F*_(1, 11)_ = 9.1; *p* < 0.012**	**1.7 s *F*_(1, 11)_ = 10.8; *p* < 0.01**	0.5 s *p* = 0.41
Fraction	7.3 ± 1.7% *p* = 0.058	−7.5 ± 2.0% *p* = 0.051	**14.8 ± 3.5% *F*_(1, 11)_ = 17.5; *p* < 0.002**	**13.3 ± 2.3% *p* < 0.007**	**−13.3 ± 2.3% *p* < 0.007**	**26.6 ± 4.4% *F*_(1, 11)_ = 36.5; *p* < 0.001**	−11.5 ± 3.2% n.s.	11.1 ± 3.3% n.s.	**22.5 ± 6.4% *F*_(1, 11)_ = 12.4; *p* < 0.005**	**11.8% *F*_(1, 11)_ = 13.3; *p* < 0.005**	7.7% *p* = 0.14

*The tests of significance used are two-tailed paired student *t*-tests (the degrees of freedom and *F*-values provided correspond to those of equivalent planned comparisons) except those marked by an asterisk which correspond to Tukey's HSD (*post-hoc* tests)*.

#### Hold /aba/ condition (no sound baseline)

The volition bias without sound is the performance difference between the *volition test* and the *passive* conditions of Experiment B (left group of bars of Figure 4 plots). When subjects were asked to hold the black lips (/aba/ viseme), the volition strength was significant for both the dominance duration [1.2 s, *F*_(1, 11)_ = 14.1; *p* < 0.005] and fraction [14.8%, *F*_(1, 11)_ = 17.5; *p* < 0.002]. *Post-hoc* tests indicate that the volition strength stemmed from significant volition biases reducing the white lips percept (/aga/ viseme) both for the dominance duration (*p* < 0.004) and fraction (*p* = 0.051) and only from a nearly significant volition bias increasing the “to hold” black lips percept (/aba/ viseme) in dominance fraction (*p* = 0.058). Subjects could successfully influence the competition dynamics and promote the black lips percept (/aba/ viseme) when watching the rivaling videos without sound.

#### Hold /aba/ condition (/aba/ voice vs. no sound)

The volition bias with the /aba/ voice is the performance difference between the hold /aba/ and the passive conditions of experiment C (middle group of bars of Figure 4 plots). When subjects were asked to hold the black lips (/aba/ viseme), the volition strength was significant for both the dominance duration [2.9 s, *F*_(1, 11)_ = 33.4; *p* < 0.001] and fraction [26.6%, *F*_(1, 11)_ = 36.5; *p* < 0.001]. Only the volition bias increasing the black lips percept dominance duration was not significant in the *post-hoc* tests, while it was on dominance fraction. This suggests that the volition strength in this condition stemmed from both a significant increase of the “to hold” /aba/ viseme dominance and a decrease of the /aga/ viseme dominance. Compared to the no sound baseline, the strength of the volitional control increased of 1.7 s for dominance durations [*F*_(1, 11)_ = 10.8; *p* < 0.01] and of 11.8% for dominance fractions [*F*_(1, 11)_ = 13.3; *p* < 0.005]. In conclusion, the capacity to promote visual lips uttering /aba/ is much improved when presented jointly with the associated /aba/ voice, supporting the idea that the more congruent sensory information is available for the perceptual competition, the stronger the volitional control will be. This finding extends that reported in van Ee et al. (2009) to higher-level BR stimuli using AV speech perception for which the contribution of sound was even more effective.

#### Hold /aga/ condition (/aba/ voice vs. no sound)

The volition bias with the /aba/ voice is the performance difference between the hold /aga/ and the passive conditions of experiment C (right group of bars of Figure 4 plots). When subjects were asked to hold the white lips (/aga/ viseme), the volition strength was again significant for both the dominance duration [1.7 s, *F*_(1, 11)_ = 9.1; *p* < 0.02] and fraction [22.5%, *F*_(1, 11)_ = 12.4; *p* < 0.005]. Nevertheless, *post-hoc* tests indicate that none of the volition biases either on the /aga/ viseme to hold or on the /aba/ viseme to suppress was by itself significant, indicating that the strength of the volitional control stemmed from a combination of both. Comparisons of the hold /aga/ condition with the no sound baseline were done under some assumptions. In order to assess whether the /aba/ voice can also improve the capacity to hold the /aga/ viseme, we compared the volition strength of this condition with that of the baseline condition without sound (hold /aba/ viseme). Although this baseline does not perfectly match the condition, we assumed that since without soundtrack the /aba/ and /aga/ viseme are visually similar, holding /aba/ or holding /aga/ viseme should not be very different. Further, since we compare volition biases—difference in performance between hold and passive conditions—even if the absolute dominance performance was to be different, we expect the relative difference to be proportional making it independent from the local baseline levels.

The limited increase of volitional control strength found when the voice was dubbed over the videos was not statistically significant: 0.5 s in dominance duration (*p* = 0.41) and 7.7% in dominance fraction (*p* = 0.14). Therefore, adding the voice /aba/ did not improve the capacity to promote the /aga/ viseme as it did for the /aba/ viseme. This finding suggests that the effect of sound on volitional control is dependent on the level of congruence between the auditory and visual information.

## Discussion

We used speech stimuli known to involve robust AV interactions in order to investigate the perceptual stabilization effect of a relevant sound on the dynamics of BR. Participants had to report continuously the dominant percept while seeing rivaling videos of mouths uttering /aba/ and /aga/. We could measure the influence of a synchronous voice saying /aba/ on the perceptual outcome either viewing passively or trying to promote a particular viseme. We evidenced different stabilization effects depending on task—passive or volition—and AV congruence—real or illusory. Since AV integration was a key aspect of this work, we decided to keep only participants highly sensitive to this McGurk combination therefore the findings reported and discussed here are only valid for this subset of the population.

### Passive effect: increased stability of dominance periods

In passive trials, we asked the participants to monitor and report continuously the color of the lips in the dominant percept, without making any effort to control the perceptual outcome. In the cases when the voice was dubbed over the rivaling talking faces, participants were told not to pay attention to it and report the visual percept just as they did in the case without sound. However, since the auditory information provided could be integrated with the visemic content carried by the lips' motion, it could indirectly interfere with perceptual dominance. Combining auditory and visual speech-related information can influence BR even passively, a similar increase in dominance duration observed for /aba/ and /aga/ viseme indicates an increased stability of the perceptual dominance periods irrespective of whether the AV matches perfectly or produces a McGurk effect. Seemingly inconsistent results have been reported in the literature concerning the effect of additional sound on the perceptual dynamics of bistable stimuli, going from a perceptual stabilization, to no noticeable effect, or to an increased alternation rate. We believe that the level of congruence between the rivaling visual percepts and the additional sound is responsible for this discrepancy, the modulation depending on the nature of the auditory stimuli. If the sound used is just an irrelevant transient sound occurring at random times during the perceptual rivalry, then it has a destabilizing effect (Takahashi and Watanabe, 2011). Instead, if the sound used can be temporally integrated with (Kang and Blake, 2005; Conrad et al., 2010) or provides semantic content to (Chen et al., 2011) one of the competing visual percepts, this congruence leads to a stabilization expressed as an increase of dominance durations depending on the degree of multimodal matching. Finally, in the case of weak or artificial multimodal integration, the stabilization effect could be so limited that it is not observable (Munhall et al., 2009; van Ee et al., 2009). In our study, although we expected to observe a reduced stabilization for the McGurk AV combination, both visemes' dominance were equally increased, which indicates that for passive observers perceived AV congruence can be sufficient to yield maximal multimodal collaboration. In both situations, the early processing of visual lips motion provides a predictive support for speech perception (Arnal et al., 2009) that speeds-up auditory processing (van Wassenhove et al., 2005). This early cross-talk seems able to cancel the exogenous shift of attention produced by the sound onset, as reported with the motion quartet illusion in Takahashi and Watanabe (2011), and allow for increased dominance durations of both percepts. If we consider the time-windows in which AV information is integrated (Baart et al., 2014), the fact that both visemes were equally stabilized suggests that this reinforcement occurs at an early stage where only spatial and temporal AV properties are integrated. We believe that the consistency between auditory and visual signals feeding the intrinsically multimodal speech processing system is a key factor in this mechanism, resulting in perceptual stabilization when relevant (Kanai and Verstraten, 2006) and in destabilization otherwise.

### Top-down attention: improved volition with congruent voice

Volitional control over the BR dynamics was tested by asking the participants to promote one of the rivaling visual percepts, holding it as long as possible when dominant and switching back to it once suppressed, this while monitoring and reporting the inevitable perceptual alternations. When the voice /aba/ was synchronously dubbed over the rivaling videos of faces uttering /aba/ and /aga/, the strength of the volitional control varied according to the type of AV congruence. The capacity of the participants to promote the physically matching /aba/ viseme was greatly improved, but not so much for the subjectively matching /aga/ viseme (that results in the illusory /ada/ auditory percept due to the McGurk effect). Although they are not aware of the AV conflict—the lips visual motion and the auditory information are always integrated—holding the /aga/ viseme, while hearing /aba/, is not equivalent to holding the /ada/ viseme. Volitional control improved greatly for real congruence resulting from physically matching AV stimulus but not so much for illusory congruence resulting from the McGurk fusion, even though phenomenal experience was similar for both (the participants being unaware of the discrepancy in the McGurk combination). Adding a synchronous looming sound improved participants' capacity to promote the looming bullseye percept that was competing in BR (van Ee et al., 2009), but two important limitations to this result were reported in this study. First, this effect is canceled if participants are not explicitly told to attend to the additional auditory modality, which was later found to prolong dominance durations even for an unrelated task (Alais et al., 2010). Second, simple synchronous tone pips were sufficient to enhance this attentional control. In this project, we tackled these limitations using natural and highly congruent AV stimulation involved in speech perception, which allowed extending the initial results of van Ee et al. (2009). We found that congruent AV robustly improved the capacity to modulate attentional control on the dynamics of visual rivalry (hold /aba/), even without explicit instruction to attend the additional voice (/aba/). Further, this effect was weaker when AV congruence was illusory (hold /aga/), which suggests that the level of congruence of the additional information influences how attentional control over BR improves (Dieter and Tadin, 2011). Finally, the size of the effects we found is substantially larger than what has been reported in the literature. This confirms the idea that if the interplay between the two modalities happens across various processing stages, which is the case for speech processing, and not limited to early visual cortex or higher brain areas, top-down attentional control has a broader range of action (Talsma et al., 2010).

### Sensory interactions in brain associative areas

**Figure 5:**
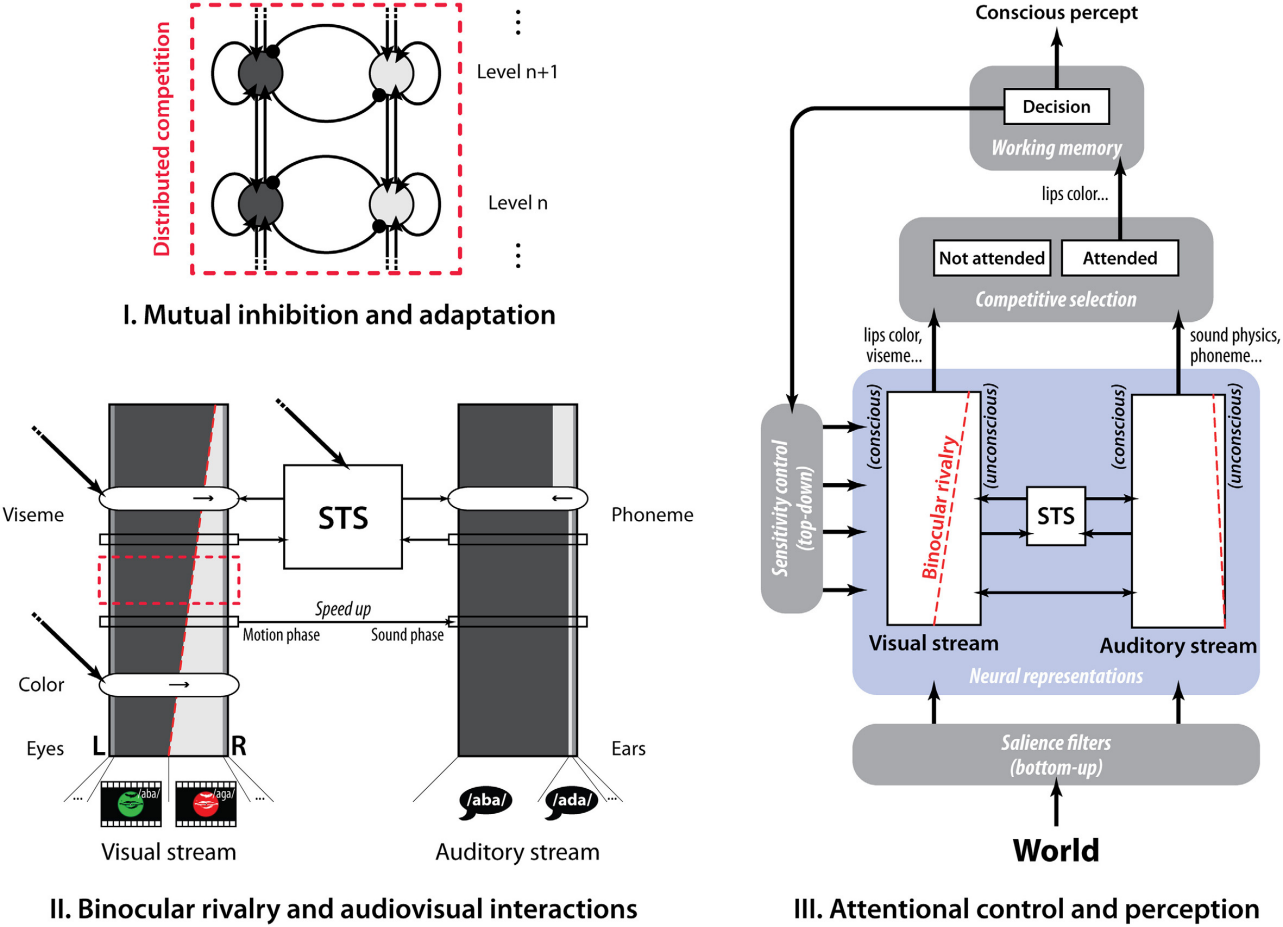
**A three-level descriptive framework based on existing models. (I)** Hierarchical view of binocular rivalry in which mutual inhibition and adaptation of the neural populations coding each perceptual state governs the competition. **(II)** Speech audiovisual interactions in STS directly modulating the binocular rivalry equilibrium. **(III)** Selective attention mechanisms (top-down sensitivity control and bottom-up saliency filters) determining the conscious outcome among the neural representations of the competing visual stimuli.

We designed our stimuli so that several features are rivaling along the visual pathway (color and lips motion), resulting in the possibility to selectively bias the competition at several levels (Dieter and Tadin, 2011). Beside, selective attention can also modulate how the voice integrates with the viseme to hold (Talsma et al., 2010), giving an additional mean to interfere with the visual competition. Based on existing models of perceptual rivalry, selective attention, multisensory integration and speech perception, we propose in Figure 5 an integrated framework of known functional interactions that provide a possible interpretation of our findings. At the lower level, BR stems from the mutual inhibition of competing features distributed along the visual pathway (Tong et al., 2006). At the intermediate level, competing visual and auditory speech information interact in brain associative areas, which modulates the strength of their neural representations. At the higher level, attention mechanisms allow promoting one neural representation through top-down sensitivity control over the competing features and bottom-up salience filters, and enables the conscious report of the winning percept (Desimone and Duncan, 1995; Knudsen, 2007).

When voice is dubbed over the talking lips, the brain tries to integrate both sensory inputs in order either to produce a consistent perceptual outcome or to dismiss the irrelevant information regarding the attended one. There is growing evidence from human fMRI and monkey single-unit recordings that the superior temporal sulcus (STS) plays an important role in AV multisensory integration. Disrupting STS with localized TMS cancels the McGurk effect, demonstrating the role played by this brain area in the visual modulation of auditory processing (Beauchamp et al., 2010). When processing AV speech, auditory and visual cortices feed information continuously into STS where signals are compared and a feedback signal is produced to modulate processing in the sensory areas (Skipper et al., 2007; Arnal et al., 2011). When both input signals are consistent, STS feedback is rapidly sent to the auditory cortex increasing its sensitivity; otherwise it takes several visual-STS-auditory loops before AV incongruence is stably inferred (Arnal et al., 2009; Baart et al., 2014). Attention modulates the sensory interplay at different levels (Fairhall and Macaluso, 2009), with a selective increase of activity observed in STS according to the congruence between voice and lip motion. Accordingly, in our study the added voice could interact with the dynamics of BR via the multisensory comparison feedback sent from STS to the visual cortex, at the level where lip motion is analyzed. At this higher processing level, neural representations are already mostly tuned to the currently dominant viseme (Leopold and Logothetis, 1999), therefore in the presence of a congruent auditory input, the AV integration feedback from STS will validate the initial visual percept thereby reinforcing its sensitivity. Nevertheless, when both visemic and phonemic inputs do not match, a greater number of neural ensembles coding for the various AV combinations is found in STS (Beauchamp et al., 2010) and the interplay with the auditory and visual cortices takes longer (several loops) before producing the fused outcome (van Wassenhove et al., 2005; Arnal et al., 2009; Baart et al., 2014). Therefore, the temporal lag introduced for McGurk fusions could be responsible for the weaker reinforcement of the corresponding percept (/aga/ viseme, illusory congruence) as compared to the quick and direct fusion (/aba/ viseme, real congruence). Regarding passive interactions, adding the voice led to a comparable increase in perceptual stability for both, suggesting that the sensitivity reinforcement from STS for perceived and real AV congruence is not a key factor and that perceptual stabilization could rely mostly on reinforcement at an early stage. Finally, in order to promote the /aba/ viseme, top-down attentional control increased the sensitivity to this stimulus at the color processing level, which biased the visual competition in favor of the percept to hold. This volitional control improved with the additional /aba/ voice when asked to hold the /aba/ viseme, but not as much when asked to hold the /aga/ viseme. The enhanced control could result from the combination of an increased sensitivity at the viseme processing level and a perceptual stabilization from AV integration in STS, the latter being selective to the targeted viseme. When holding /aga/ viseme, although eventually perceived congruent with the /aba/ phoneme, STS feedback would arrive later and be less precise for McGurk AV combinations, limiting the top-down sensitivity controls and STS stabilization (Arnal et al., 2009; Baart et al., 2014).

## Conclusions

We investigated visual competition and top-down modulation by selective attention using speech stimuli known to involve interactions between the visual and auditory cortices at various levels. The study of this phenomenon is fundamental as it directly addresses the extent to which human will can determine the conscious visual outcome among several possible ones. We measured the effect of an additional voice on the competition dynamics of talking lips manipulating the type of AV congruence for participants highly sensitive to the McGurk effect (41.4% of our initial set). First, when observing passively the rivaling lips motion, adding the voice /aba/ increased similarly the dominance of the physically congruent /aba/ viseme and of the illusory congruent /aga/ viseme. This indicates that subjectively built matching is enough for perceptual stabilization. Second, adding the voice /aba/ increased the capacity to promote the physically congruent /aba/ lips, but not so much the illusory congruent /aga/ lips, although it could be equivalent to promoting lips uttering /ada/. This finding demonstrates that a perfectly congruent sound enhances considerably attentional control over the selection mechanism underlying the perceptual outcome. On the other hand, it seems that a higher level of auditory congruence is necessary to increase significantly how the top-down attentional modulation interferes with the perceptual competition, despite the fact that real and illusory congruence generate qualitatively similar phenomenal conscious experiences of the multimodal input. Finally, stabilization differences for passive viewing and volition according to the type of AV congruency suggest there are at least two distinct processes at stake (STS feedback reinforcement and top-down sensitivity control) which can be differentiated in terms of time-window of integration (early vs. later stage). We proposed a unified framework that accounts for these findings and those previously reported.

### Conflict of interest statement

The authors declare that the research was conducted in the absence of any commercial or financial relationships that could be construed as a potential conflict of interest.
