# Supplementary material for: Hearing (rivaling) lips and seeing voices: how audiovisual interactions modulate perceptual stabilization in binocular rivalry
Source: Front Hum Neurosci. 2014 Sep 4;8:677. doi: 10.3389/fnhum.2014.00677 (PMC4154468; doi:10.3389/fnhum.2014.00677)
Supplement: Supplementary file 1 [file DataSheet1.DOCX]

***Supplementary Material***

**Hearing (Rivaling) Lips and Seeing Voices: How Audiovisual Interactions Modulate Perceptual Stabilization in Binocular Rivalry**

**Manuel Vidal^1,2^* and Victor Barrès^1^**

^1^Laboratoire de Physiologie de la Perception et de l’Action, UMR7152 Collège de France & CNRS, Paris, France

^2^Institut de Neurosciences de la Timone, UMR7289 Aix-Marseille Université & CNRS, Marseille, France

*** Correspondence:** Manuel Vidal, Institut de Neurosciences de la Timone, Campus santé Timone, 27 boulevard Jean Moulin, 13385 Marseille cedex 5, France

manuel.vidal@univ-amu.fr

# Preliminary experiment: selecting McGurk sensitive subjects

A recent meta-analysis proved that the large variability observed in McGurk AV fusion stems from large individual differences, some participants being more “visual” and others more “auditory” (Schwartz 2010). In this project, AV correspondence is a key factor for the hypothesis we test, therefore we designed a preliminary experiment to select the participants for whom the lips motion and the voice used later in the main experiments are never perceived as incongruent. In this preliminary experiment, we assessed subjects’ individual sensitivity to the McGurk and MacDonald effect (1976) in which a voice saying /aba/ synchronously dubbed over a video of lips uttering /aga/ results in an audiovisual (AV) fusion producing the illusory auditory percept /ada/. Trials consisted of a single AV presentation lasting 1.64s followed by a forced choice task (see the upper-left panel of **Figure 2** from the article). Videos were presented binocularly (no rivalry here) and consisted either in lips uttering /aba/ or lips uttering /aga/, with always the same /aba/ soundtrack. In the forced choice, subjects had to select between /aba/ and /ada/, which one was the closest to the auditory percept they just heard. Subjects were instructed to look at the fixation cross while keeping their attention focused on both the lip motion and the sound, and then to answer quickly and instinctively. When presented with the /aga/ video, subjects fully sensitive to the McGurk effect would always select /ada/, whereas those insensitive would always select /aba/. The experiment was composed of 4 blocks of 20 trials. In each block, 10 trials used the lips uttering /aba/ and the other 10 the lips uttering /aga/. The color filtering of the videos and lips motion were randomized by groups of 4 conditions representing the 4 possible combinations. A short break after each block was proposed. Subjects started with a 16 trials training session, which used the same experimental conditions. The entire experiment lasted about 10 minutes.

For each subject, we computed the sensitivity to the McGurk effect (from 0% to 100%) with the following ratio: the proportion of /ada/ heard when lips uttering /aga/ were shown divided by the proportion of /aba/ heard when lips uttering /aba/ was shown. The numerator represents the proportion of AV fusion as expected by the McGurk effect. The denominator corrects for lapsing errors, it was equal to one for most of the subjects. From the 29 subjects tested, we excluded 17 whose McGurk effect was below the minimal criterion of 87.5%. The selected group included 12 subjects for whom AV fusion occurred almost systematically (individual effect above 87.5%, group mean 97.0% ± 3.3%). Contrarily to the population tested by McGurk and MacDonald for which this AV combination resulted in a very robust illusion, in the French student population more than half are simply not sensitive to it (see the statistics reported in Cathiard, Schwartz, and Abry 2001).

# Perceptual report correction rules

The perceptual dominance reports were corrected to remove initial and final reports, to suppress very short piecemeal durations reported between two identical perceptual states, and to adjust successive dominance durations when overlapping. We used the three following three rules for these corrections:

**Border effects.** Perceptual reports during the first 10 seconds of stimulus presentation were not taken into account, these being often messy with lots of piecemeal. Furthermore, differences exist between the initial transitory regime of binocular rivalry and the following sustained oscillatory regime that we are interested in (Carter and Cavanagh 2007). The first percept analyzed was the first starting after this initial period and the last percept analyzed was the last that fitted entirely before the end of the 130s (i.e. the last that ended due to a change in perceptual state).

**Two consecutive equal percepts.** In the case when the subject went from one percept and back to the same percept after a duration *d* with both keys released (signaling a mixed percept), if *d* was shorter than 500ms the mixed duration was not taken into account and the dominance duration of the percept included both durations and the mixed duration.

**Overlap of percept report.** When going from one percept to another, if the second percept report started (associated button pressed) before the first one ended (associated button released) then the first percept duration was shortened so to remove the overlapping period.

# Checking our stimuli on BR fundamental properties

In this appendix, we checked whether the three characteristics of BR – exclusivity, a standard stochastic component and inevitability – hold for the static and animated rivaling stimuli created for this project. We also controlled for the good balance according to the color filtering and for the uniform probability of perceptual switches within the video sequences.

## Static image rivalry (exp. A)

Experiment A was designed for three purposes. The first was to ensure that our participants could correctly experience BR with our setup that used an HMD instead of the traditional stereoscope. For both gratings and static lips conditions, a low proportion of ambiguous or mixed percept was reported which indicates that the rivaling percepts are mutually exclusive (exclusivity). The piecemeal level of our rivaling lips (dominance fraction mean ± SE: 9.51 ± 1.88%) was slightly lower than with the gratings (11.26 ± 2.83%). The gamma shaped distribution of dominance durations observed for each condition and the standard level of sequential correlation between successive dominance durations observed are compatible with typical BR (see paragraph A.3.3 below). Finally, subjects could not prevent the perceptual alternations from one image to the other (inevitability). We can conclude that our setup as well as the static version of rivaling lips did produce the standard BR phenomenon.

The second purpose was to measure individual oscillatory dynamics to serve as a baseline for the following experiments. Dominance durations with the static lips (3.66 ± 0.51s) were slightly longer though not significantly for the black lips percept (4.19 ± 0.70s) than for the white lips percept (3.31 ± 0.43s). Dominance fractions confirmed this trend with 51.7% against 39.2%, respectively, suggesting that the black lips stimulus is stronger than the white lips.

The third purpose was to check with the simulated rivalry condition if subjects dutifully reported the alternations and to measure the individual reaction times. Ten subjects reported correctly 100% of the percepts and the other two were above 95% of correct responses, resulting in an average of 99.4 ± 0.42%. This shows that subjects were perfectly able to report what they saw and performed the task correctly. The average reaction time measured with this condition was 448 ± 22ms.

## Video rivalry (exp. B and exp. C)

Designing rivaling video stimuli was the main technical challenge of this project, and the results from experiment B and C proved that we were successful. For both the passive and volition test conditions, a low proportion of mixed percept was reported (exclusivity, with 10.2 ± 4.1% and 10.4 ± 3.7% of piecemeal respectively). Dominance durations in all conditions followed a gamma shaped distribution and showed a low level of sequential correlation that is compatible with classical BR stimuli. Subjects could not prevent perceptual alternations even when instructed to in the volition test conditions (inevitability). The three characteristics of binocular rivalry still hold for our pairs of rivaling videos of talking lips, which proves that with proper color filtering it is possible to use natural videos to produce competing stimuli in binocular rivalry. Finally, motion being known to trigger perceptual switches, we checked whether some motion features of the video sequences could significantly trigger perceptual switches and thus interfere with the natural stochasticity of dominance durations. We also verified that the presence of sound in experiment C did not unduly disturb the dynamics by triggering switches (see §3.4 below).

## Distribution of dominance durations

A standard level of stochastic component of dominance durations is one of the three fundamental characteristics of BR. In this section, we analyzed on the one hand the shape of the dominance duration distribution and on the other hand the level of sequential correlations between two successive identical percepts. In **Supplementary** **Supplementary Figure 1** we plotted the normalized dominance duration distribution of 4 representative experimental conditions: gratings rivalry **(A)**, static lips rivalry **(B)**, videos of lips rivalry without sound in the passive condition **(C)** and in the volition test condition **(D)**. Normalized dominance durations were computed for each subject as the dominance duration divided by the average yielded in the considered condition. These normalized durations were then grouped in bins that started from 0 and increased in steps of 0.2s. These individual distributions were finally averaged across all subjects so that each of them contributes equally to the final distribution. We then fitted each histogram with a gamma distribution function (red dashed lines) and computed the corresponding root mean square errors (ε) to indicate the goodness of the fits.


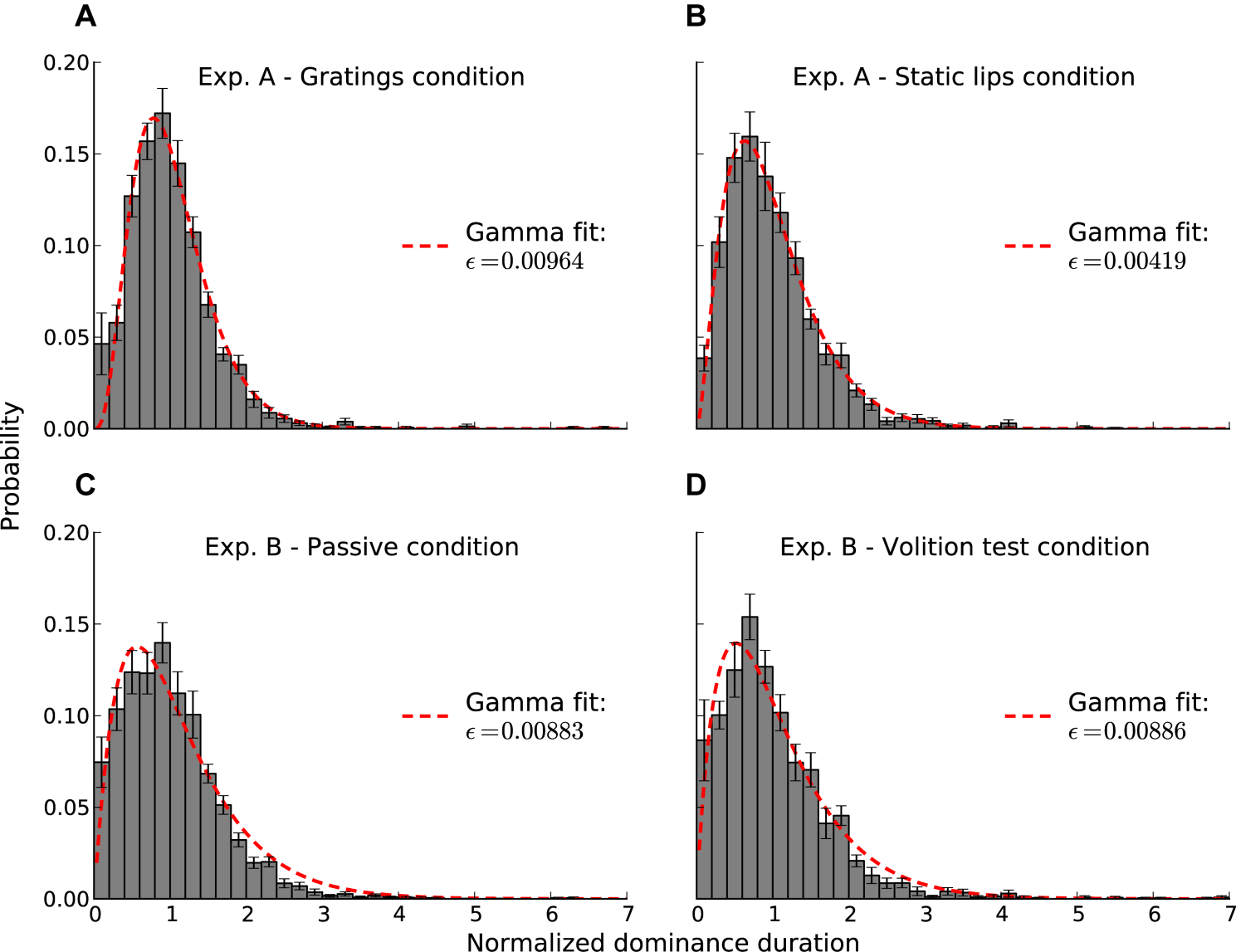


**Supplementary** **Figure 1. Normalized dominance duration distributions for four conditions:** the gratings **(A)** and static lips **(B)** conditions from experiment A and the passive **(C)** and volition test **(D)** conditions from experiment B. Each histogram was fitted with a gamma distribution function (red dashed lines) and the corresponding root mean square errors (ε) were computed in order to indicate the goodness of the fits. Error bars represent the standard error of the mean.

Additionally, we carried out sequential correlation analyses as described in (van Ee 2009) for the same 4 conditions. For each subject, we calculated the Spearman’s rank correlation (rho) between the dominance duration of a particular percept with the immediately following dominance duration of the same percept (denoted by lag 1). **Supplementary** **Supplementary Table 1** gives for each experimental condition the rho value averaged over subjects and the p value associated with the Spearman Rank Correlation test. We found for gratings a mean correlation of rho=0.17, which is slightly below the rho=0.2 value reported in (van Ee 2009) with an identical condition. Importantly, similar values are found for static lips rivalry (rho=0.16), video rivalry without sound in the passive condition (rho=0.22) and in the volition test condition (rho=0.15). Indeed, paired t-test did not yield any significant difference between these correlation values. We can therefore conclude that the stochastic component of perceptual alternation dynamics observed for our novel video rivalry stimuli is perfectly in line with what is usually found for classical BR stimuli.

**Supplementary** **Table 1. Sequential correlation analyses.** The Spearman’s rank correlation (rho) between the dominance duration of a particular percept with the immediately following dominance duration of the same percept (denoted by lag 1).

| Spearman’s rank correlation | |  | rho |  | p |
| --- | --- | --- | --- | --- | --- |
| Exp. A | Gratings |  | 0.1659 |  | 7.02e-06 |
|  | Static lips |  | 0.1574 |  | 5.26e-04 |
| Exp. B | Passive |  | 0.2222 |  | 1.00e-07 |
|  | Volition test |  | 0.1512 |  | 5.69e-04 |

## Distribution of perceptual switches

Motion being known to trigger perceptual switches, we checked whether some motion features of our video elementary sequences could generate significantly more switches and thus interfere with the natural stochasticity of dominance durations. We analyzed the repartition of switch occurrences in the looping 1640ms elementary video sequence. For each subject, perceptual switches were pooled together in time bins of 80ms ranging from 0 to 1640ms. **Supplementary** **Supplementary Figure 2** shows the switch probability distributions averaged across all subjects and all conditions of experiment B (without voice) and C (with the synchronized /aba/ voice). The proportion of switches that occurred in each time bin was then compared to the probability level expected for a uniform probability distribution (dashed lines in the plots) using t-tests against single value for each bin. Only one time bin of the stimuli without sound was significantly different than the expected average value at about 400ms (indicated by the asterisk). This indicates that in our experiments, the lips’ motion per se had no effect on the perceptual competition dynamics.


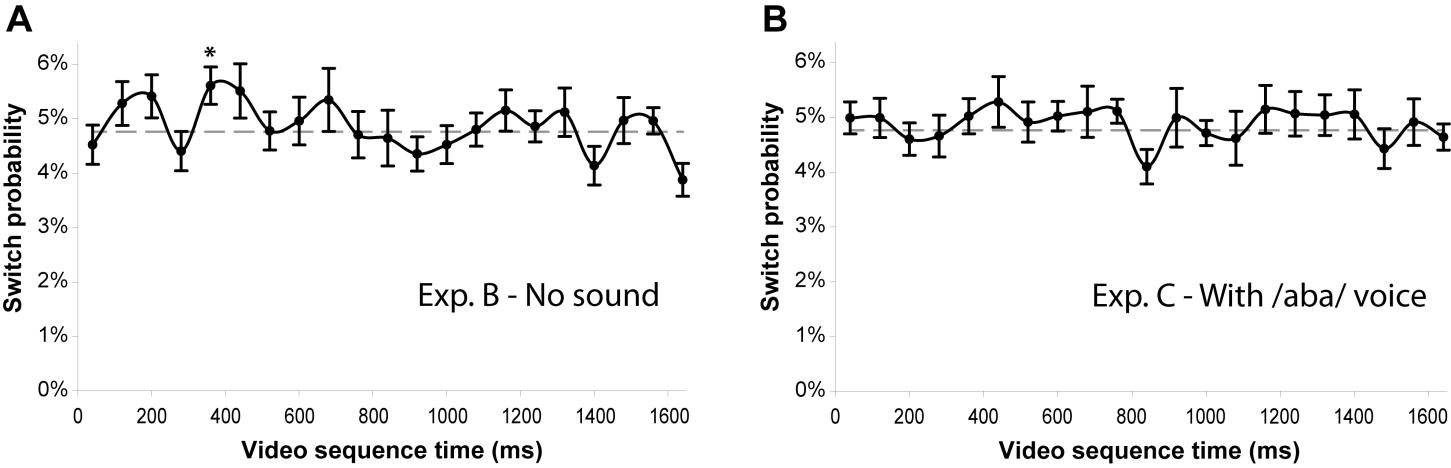


**Supplementary** **Figure 2. Switch probability distribution along the 1640ms looping video sequence.** For the no sound condition **(A)** from experiment B and the /aba/ voice condition **(B)** from experiment C. The dashed-lines show the probability level expected for a uniform probability distribution. Error bars represent the standard error of the mean.

## Color filtering: controlling for good balance

The isoluminance of the rivaling stimuli (black lips on a green background vs. white lips on a red background) was matched based on the first image of the video sequences in which the mouth was closed. This was done in order to prevent large differences in intensity to bias the competition for dominance in favor of one of the color filtering, however it is necessary to check a posteriori whether this was successful. **Supplementary** **Supplementary Table 2** shows the dominance durations and fractions of the passive condition without sound for each color filtering. The very limited differences observed between black and white lips were never statistically significant (student t-test): neither when visemes and color association were balanced (for passive effects) nor when they were not (for volitional control). We can conclude that the two color associations result in similar intensity regarding the rivalry. Therefore, the effects reported here are independent of the viseme/color association and stem only from the different interactions between the voice and the lips’ motion.

**Supplementary** **Table 2. Control for passive effects in which visemes were balanced and for volition effects in which visemes were not balanced.** Dominance durations and fractions are not significantly different which indicates that the two color filtering used could not have biased the results.

|  | Control for passive effects (visemes balanced) | | |  | Control for volition effects (visemes not balanced) | | |
| --- | --- | --- | --- | --- | --- | --- | --- |
|  | Black lips | White lips | Difference |  | Black lips | White lips | Difference |
| Duration | 4.03 ± 0.66s | 3.88 ± 0.63s | 0.148s (p=0.66) |  | 3.92 ± 0.65s | 4.29 ± 0.72s | -0.36s (p=0.98) |
| Fraction | 48.0 ± 4.1% | 45.7 ± 4.1% | 2.3% (p=0.77) |  | 44.3 ± 4.2% | 45.5 ± 4.3% | -1.2% (p=0.87) |

# References

Carter, Olivia, and Patrick Cavanagh. 2007. “Onset Rivalry: Brief Presentation Isolates an Early Independent Phase of Perceptual Competition.” *PLoS ONE* 2 (4): e343. doi:10.1371/journal.pone.0000343.

Cathiard, M. A., J. L. Schwartz, and C. Abry. 2001. “Asking a Naive Question about the McGurk Effect: Why Does Audio [b] Give More [d] Percepts with Visual [g] than with Visual [d]?” In *AVSP 2001-International Conference on Auditory-Visual Speech Processing*. http://www.isca-speech.org/archive_open/avsp01/av01_138.html.

McGurk, H., & MacDonald, J. (1976). Hearing lips and seeing voices. *Nature*, *264*(5588), 746-748.

Schwartz, Jean-Luc. 2010. “A Reanalysis of McGurk Data Suggests That Audiovisual Fusion in Speech Perception Is Subject-Dependent.” *The Journal of the Acoustical Society of America* 127 (3): 1584–94. doi:10.1121/1.3293001.

Van Ee, R. 2009. “Stochastic Variations in Sensory Awareness Are Driven by Noisy Neuronal Adaptation: Evidence from Serial Correlations in Perceptual Bistability.” *Journal of the Optical Society of America. A, Optics, Image Science, and Vision* 26 (12): 2612–22.
